# Prognostic value of preoperative neutrophil-to-lymphocyte and platelet-to-lymphocyte ratios, and multiphasic renal tomography findings in histological subtypes of renal cell carcinoma

**DOI:** 10.1186/1471-2490-14-95

**Published:** 2014-11-26

**Authors:** Suat Keskin, Zeynep Keskin, Hakan Hakki Taskapu, Havva Kalkan, Mehmet Kaynar, Necdet Poyraz, Hatice Toy

**Affiliations:** Department of Radiology, Necmettin Erbakan University, Meram School of Medicine, Beyşehir Street, Akyokuş, Meram, Konya, 42080 Turkey; Department of Radiology, Konya Training and Research Hospital, Konya, Turkey; Department of Urology, Necmettin Erbakan University, Meram School of Medicine, Konya, Turkey; Department of Urology, Selçuk University, School of Medicine, Konya, Turkey; Department of Pathology, Necmettin Erbakan University, Meram School of Medicine, Konya, Turkey

**Keywords:** Renal cell carcinoma, Neutrophil-to-lymphocyte ratio, Platelet-to-lymphocyte ratio, Multiphasic multidetector tomography, Mortality

## Abstract

**Background:**

To determine the relationship between renal cell carcinoma subtypes and the associated mortality and biochemical parameters. An additional aim was to analyze multiphasic multidetector computed tomography findings.

**Methods:**

This study is a hospital-based retrospective investigation, using 211 patients with a diagnosis of renal cell carcinoma upon computed tomography examination. The histological subtypes included clear cell in 119 patients, chromophobe cell in 30 patients, papillary cell in 25 patients, mixed cell in 32 patients, and sarcomatoid cell in 4 patients.

**Results:**

The mean age of the patients participating in this study was 61.18 ± 11.81 years, and the mortality rate was 10.4% (n = 22) through the 2-year follow-up. The ratios of both the neutrophil-to-lymphocyte upon admission to the hospital and platelet-to-lymphocyte of the non-surviving group were significantly higher than those of the surviving group (p < 0.05). When the analysis of the 2-year survival of the patients was examined according to the median platelet-to-lymphocyte ratio values, the Kaplan-Meier survival curves were significantly different between the surviving and non-surviving groups (p = 0.01). In two-way analysis of variance test, statistically significant results which were influenced by mortality (p = 0.028) and were found between renal cell carcinoma subtypes in the computed tomography density of corticomedullary phase (p = 0.001).

**Conclusions:**

The neutrophil-to-lymphocyte ratio and platelet-to-lymphocyte ratio may represent widely available biomarkers in renal cell carcinoma, and the logistic regression model indicated that neutrophil-to-lymphocyte ratio was a significant predictor for mortality. According to the median platelet-to-lymphocyte ratio values, the Kaplan-Meier survival curves were significantly different between the surviving and non-surviving groups.

## Background

Renal cell carcinoma (RCC) is the most common primary renal tumor, and originates from the renal cortex [1]. Worldwide, the incidence of RCC has increased over the last thirty years [2]. After the surgical treatment of RCC, the possibility of recurrent tumors is 10-20% [3–5] therefore, with the recent peripheral blood sampling in the diagnosis of RCC, the development of different prognostic factors is necessary. For this purpose, some prognostic markers and models have been developed; [6, 7] for example, changes like neutrophilia, lymphopenia, and thrombocytosis in the peripheral blood are evaluated in response to systemic inflammation. Prognostic factors, such as the neutrophil-to-lymphocyte ratio (NLR) and platelet-to-lymphocyte ratio (PLR), are used for an assessment of the response to systemic inflammation and RCC prognosis [8–11]. Various studies have shown that a high NLR is associated with poor prognosis in RCC; however, in most of these studies, clear cell, non-clear cell, metastatic, and non-metastatic RCC groups were included [11–14]. The relationship of the histological RCC subtypes with the prognostic factors, such as NLR, PLR, or mortality, has not been actively discussed in the literature. In this study, clear cell/non-clear cell or metastatic/non-metastatic differentiation has been made. Also, multiphasic multidetector computed tomography (CT) findings are included, in addition to the hematological parameters. Most commonly used imaging modality in the detection of renal masses is multidetector CT. Many studies in the past reported significant findings that revealed the different enhancement patterns of RCC subtypes on multidetector CT [15–17].

## Methods

### Study population

This study is a hospital-based retrospective investigation, where 231 patients with RCCs over the age of 18, who were admitted to the Meram Medical School Hospital, were screened between January of 2008 and March of 2013, were enrolled in the study after providing written informed consent. Patients with a diagnosis of RCC upon computed tomography (CT) examination were included in the study. The RCCs were diagnosed by resection, and 20 patients without pathological correlation were excluded from the study population. This retrospective study was approved by the Necmettin Erbakan University, Meram School of Medicine Hospital Institutional Review Board.

### Study protocol

The RCC cases were screened from the database of the hospital’s electronic record system. The demographic and clinical characteristics of 211 patients who met the inclusion criteria were obtained from the patient’s archived records to evaluate mortality. RCCs were detected via multiphasic multidetector CT, and hospital mortalities of the patients were determined according to their medical records. Fuhrman grade system was used for pathological grading in RCC.

### Blood sample analysis

A complete blood count analysis was performed using the peripheral venous blood samples taken upon admission to the urology department of the hospital. The blood samples were collected in a calcium EDTA (Ethylenediaminetetraacetic acid) tube, and blood counts have been evaluated using an auto-analyzer (Abbott Cell-Dyn Ruby Hematology Analyzer, Illinois, USA) in our hospital since 2007. The NLR was calculated as the ratio of neutrophils to lymphocytes, and the PLR was calculated as the ratio of platelets to lymphocytes in the peripheral blood. In addition, other routine laboratory findings were examined using the digital record systems of the hospital.

### Equipment and scanning

One radiologist (SK), with 9 years of experience in CT, carried out the multiphasic multidetector CT examinations. All of the patients were examined using a 64-multi-detector CT (64-MDCT) scanner (Siemens SOMATOM Sensation 64, Erlangen, Germany) with the following scanning parameters: 0.6 mm collimation, 1.5 mm slice thickness, 1.4 mm increment, 100 kV and 135 mAs, a pitch of 0.9, and a gantry rotation time of 0.33 s. A scout image was acquired while the patient was in the supine position, and the area from the diaphragm to the end of the iliac bones was identified as the field of examination. All patients received 100 ml of nonionic contrast medium (Ultravist 300; Bayer Schering Pharma, Berlin, Germany), which was managed through a catheter in the right antecubital vein. The contrast was executed at a flow rate of 5 mm/sec using an automated injector, and the scan was executed 20 s after the start of the injection. Firstly, unenhanced phase was obtained. After the nonionic contrast medium was given, the corticomedullary phase in the 25^th^ second, nephrographic phase in the 60^th^ second, and excretory phase in the 5^th^ minute were obtained. Lesion sizes were measured in the nephrographic phase and densities were measured in the unenhanced, corticomedullary, nephrographic and excretory phases. Density measurements were made from the 3 separate 10 mm^2^ solid circular region of interest (ROI) field in the mass and then the average of these values were calculated for the mass attenuation. The most homogeneous and dense contrast enhanced areas were used to obtain the attenuation values. In contrast, we avoided from necrotic, cystic and calcified areas to measure the density.

### Statistical analysis

Statistical analyses were performed using SPSS version 20.0 software (SPSS, Chicago, IL, USA), and the data were presented as the mean ± standard deviation or median (interquartile range [IQR]). Subsequently, the lesion sizes were categorized as less than 40 mm, 41–80 mm, 81–120 mm, 121–160 mm, and 161–200 mm. Distribution normality was analyzed with the Kolmogorov-Smirnov test, and the differences between the two groups were tested using the independent Student’s *t*-test for normally distributed variables, while the Mann–Whitney U test was used for a comparison of the non-parametrically distributed variables. The differences between the categorical variables were determined by using the χ2-test. While investigating the associations between non-normally distributed and/or ordinal variables, the correlation coefficients and their significance were calculated using the Spearman test. One-way analysis of variance was used to compare CT density among the subtypes of RCCs. Levene’s test was used to assess the homogeneity of the variances. When an overall significance was observed, pair wise post-hoc tests were performed using Tamhane’s T2 test. When investigating the changes in lesion density of corticomedullary phase by RCC’s subtypes, the effects of mortality was adjusted using two-way analysis of variance test respectively. Kruskal-Wallis tests were conducted to compare the differences in CT density and NLR among the pathological subtypes, and hemoglobin and creatinine among lesion sizes and tumor stage. The Mann–Whitney U test was performed to test the significance of the pair-wise differences using the Bonferroni correction to adjust for multiple comparisons. Two-tailed *P* values of less than 0.05 were accepted to be statistically significant. The logistic regression analysis was performed to determine the predictors of 2-year mortality. The hematological parameters, age, gender, NLR, and PLR were included in this regression model as independent variables. The best cut-off value of the hematological parameters was estimated using regression tree analysis.

## Results

**Figure 1:**
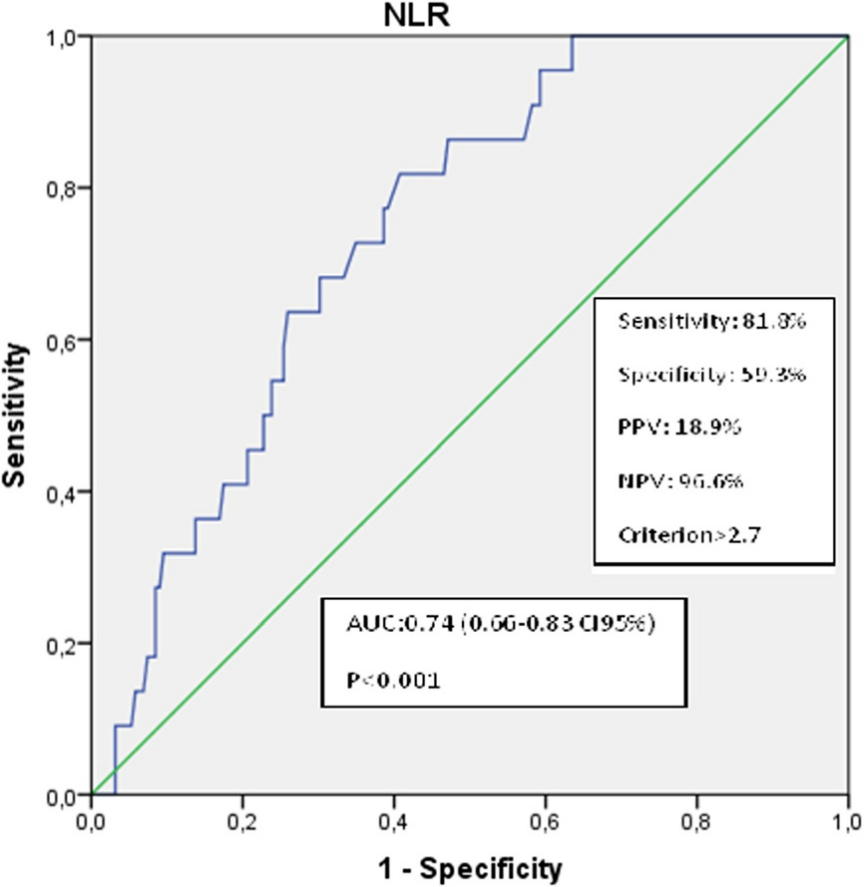
**ROC curve showed predictive value of NLR for death.**

**Figure 2:**
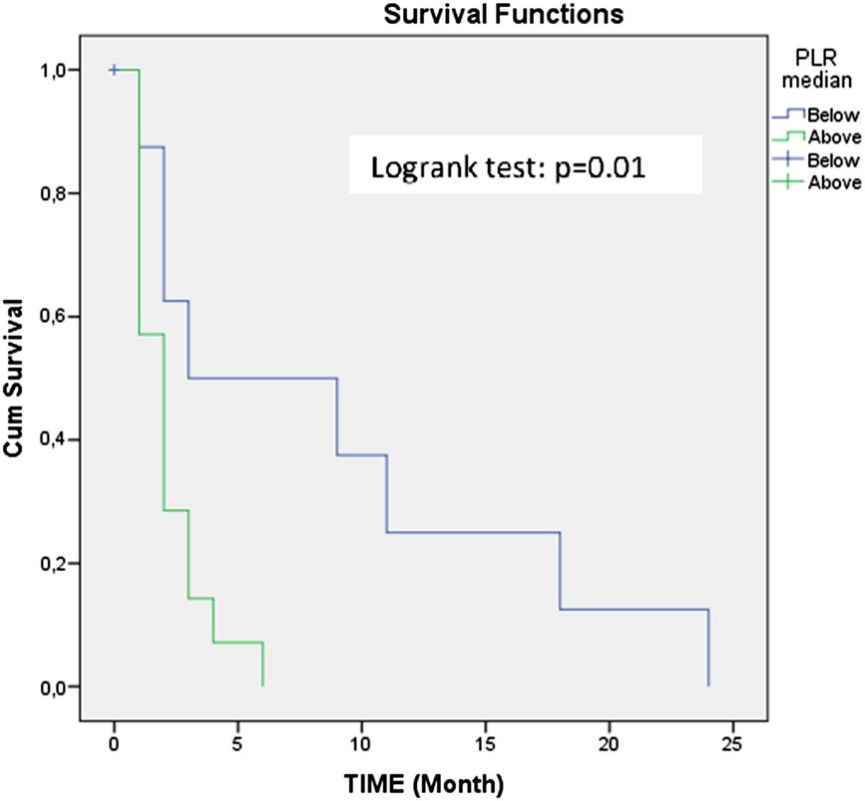
**Kaplan Meier survive curve according to the PLR median value.**

The mean age of the 211 patients who were diagnosed with renal cell cancer was 61.18 ± 11.81 years, and the mortality rate was 10.4% (n = 22) by the 2-year follow-up. The histological subtype was clear cell carcinoma in 119 patients, chromophobe cell carcinoma in 30 patients, papillary cell carcinoma in 25 patients, mixed cell carcinoma in 32 patients, and sarcomatoid cell carcinoma in 4 patients. The mean and 95% CI of CT density were demonstrated in subtypes of RCCs (Table 1). Demographic and laboratory findings of the surviving and non-surviving patients are reported in Table 2. Both the NLR upon admission to the hospital and the PLR of the non-surviving group were significantly higher than those of the surviving group (p < 0.05). Also, there were significant differences in age, lesion size, WBCs, neutrophils, lymphocytes, platelets, hemoglobin, creatinine, metastasis, and tumor stage (Table 2). Age, NLR, hemoglobin, and creatinine were independent predictors of mortality in the logistic regression analysis (Table 3). The optimal cut-off value for NLR as a predictor of mortality was determined to be 2.7 in the ROC curve analysis. On this level, the sensitivity was 81.8%, specificity was 59.3%, positive predictive value was 18.9%, and the negative predictive value was 96.6% (Figure 1). There is no significant difference in NLR between pathological subtypes (p = 0.928). The optimal cut-off value for the PLR was determined to be 151. When the analysis of the 2-year survival of the patients was examined according to median PLR values, the Kaplan-Meier survival curves were significantly different between the surviving and non-surviving groups (p = 0.01) (Figure 2). In the Mann–Whitney U test, statistically significant results were found in the WBCs (p = 0.021), platelets (p = 0.003), hemoglobin (p < 0.001), lesion density of excretory phase (p = 0.048) and creatinine (p < 0.001) between the male and female patients; the PLR (p = 0.04) and lesion density of corticomedullary phase (p = 0.001) between the clear cell and non-clear cell lesions; and the lesion size (p < 0.001), creatinine (p < 0.001), and hemoglobin (p = 0.001) between metastasis and non-metastasis. When the males and females were compared, there was a statistically significant difference in the metastasis between the two groups (p = 0.02). In the Kruskal-Wallis test, there was a statistically significant difference in corticomedullary phase’s density between the clear cell and papillary (p < 0.001), chromophobe and papillary (p = 0.016), and papillary and mixed (p = 0.002) RCCs. In the Kruskal-Wallis test, there was a statistically significant difference in hemoglobin and creatinine between stage 1 and 4 (p < 0.001). In the independent Student’s *t*-test, the lesion’s density in corticomedullary phase was found 21,12 HU higher than men in women (p = 0.034 CI%95 1,59-40,65), the lesion’s density in corticomedullary phase was found 27,34 HU higher than non-clear cell RCCs in clear cell RCCs (p = 0.006) (CI%95 8,16-46,53), the lesion’s density in nephrographic phase was found 19,73 HU higher than non-clear cell RCCs in clear cell RCCs (p < 0.001) (CI%95 9,04-30,42) and the lesion’s density in corticomedullary phase was found 29,53 HU higher than non-surviving in surviving group (p = 0.048) (CI%95 0,23-58,47). In the One-way analysis of variance test, statistically significant results were found between clear cell and papillary (p < 0.001), clear cell and sarcomatoid (p < 0.001), papillary and mix (p = 0.046), papillary and sarcomatoid subtypes (p < 0.031) in the lesion density of early arterial phase. Statistically significant results were found between clear cell and papillary (p = 0.001), papillary and mix (p = 0.039) in the lesion density of nephrographic phase. In Spearman test, positive medium correlation (r 0.40-0.60) was found between unenhanced and corticomedullary phases. Positive good correlation (r 0.60-0.70) was found between unenhanced and corticomedullary phases. Positive good correlation (r 0.60-0.70) was found between corticomedullary and nephrographic phases. Positive excellent correlation (r 0.75-1.00) was found between nephrographic and excretory phases (p < 0.05). In two-way analysis of variance test, statistically significant results which were influenced by mortality (p = 0.028) and were found between RCCs subtypes in the CT density of corticomedullary phase (p = 0.001).

**Table 1:** **The mean and 95% CI of CT density in subtypes of RCCs**

	Clear cell RCC		Chromophobe RCC		Papillary RCC		Mixed RCC		Sarcomatoid RCC	
	Mean	95% CI	Mean	95% CI	Mean	95% CI	Mean	95% CI	Mean	95% CI
unenhanced	31,43 ± 1,50	28,41-34,45	38,25 ± 5,35	25,60-50,90	31,70 ± 3,81	23,08-40,32	33,78 ± 3,42	25,90-41,65	29,50 ± 1,50	10,44-48,56
corticomedullary	118,24 ± 5,83	106,47-130,01	117,38 ± 18,21	74,33-160,42	58,20 ± 5,21	46,41-69,99	106,33 ± 12,49	77,52-135,15	79,00 ± 1,00	66,29-91,71
nephrographic	92,83 ± 2,78	87,23-98,44	88,13 ± 12,54	58,47-117,78	55,10 ± 6,24	40,99-69,21	82,56 ± 5,38	70,15-94,96	60,50 ± 9,50	60,21-181,21
excretory	66,62 ± 2,42	61,74-71,50	63,00 ± 9,45	40,66-85,34	51,70 ± 7,23	35,33-68,07	66,56 ± 5,62	53,61-79,50	47,50 ± 4,50	9,68-104,68

*RCC*: Renal cell cancer, *CI:* Confidence interval, *CT:* Computed tomography.

**Table 2:** **Demographics and laboratory findings of survived and non-survived patients**

	Survived (n = 189) mean ± SD/median (IQR)	Non-survived (n = 22) mean ± SD/median (IQR)	P	Z
Age years	58.1 ± 13.5	67.9 ± 12.5	0.005	
Gender (F/M)	69/120	7/15	0.665	
Lesion size	56.9 ± 30.6	75.2 ± 35.2	0.004	-2.853
WBC (10^3/uL)	7.89 (7.63)	11.96 (10.30)	0.002	-3.075
Neutrophil (10^3/uL)	5.09 (4.92)	8.50 (7.71)	0.002	-3.033
Lymphocyte(10^3/uL)	2.13 (2.01)	2.08 (2.10)	0.457	-0.744
NLR	2.60 (2.24)	4.28 (3.75)	<0.001	-3.733
Platelet (10^3/uL)	259.0 ± 73.1	306.1 ± 65.9	0.028	-2.203
PLR	134.2 (124.0)	150.2 (145.0)	0.038	-2.079
Hemoglobin mg/dl	13.0 ± 1.8	12.4 ± 1.6	0.001	-3.371
MPV	7.24 (7.20)	7.13 (7.86)	0.430	-0.790
Creatinine mg/dl	0.9 ± 0.3	1.2 ± 0.8	0.046	-1.996
CT density (HU)				
Unenhanced	32.9 ± 1.4	29.7 ± 3.1	0.401	
Corticomedullary	110.8 ± 5.3	81.4 ± 10.1	0.048	
Nephrographic	86.5 ± 3.1	73 ± 5.3	0.118	
Excretory	64.2 ± 2.5	59 ± 4.4	0.522	-0.640
Metastasis	44	12	0.002	9.88 chi-square
Pathologies Clearcell/Nonclear cell	103/86	16/6	0.103	
Chromophobe/ Papillary/Mix/Sarcomatoid	29/25/29/2	1/0/3/2	0.692	
Tumor Stage 1/2/3/4	105/27/13/44	7/1/2/12	0.005	-2.788

*IQR*: interquartile range, *WBC:* White blood cell count, *NLR:* Neutrophil-lymphocyte ratio, *PLR:* Platelet-lymphocyte ratio, *MPV:* Mid-platelet volume, *CT:* Computed tomography, *HU:* Hounsfeld unit.

**Table 3:** **Logistic regression results for the predictors of mortality**

Variables	Odds ratio	95.0% CI HR	P
***The first step***			
Age	1.05	1.01-1.10	0.015
Gender	0.96	0.35-2.59	0.93
NLR	3.21	1.26-8.16	0.014
PLR	1.79	0.60-5.30	0.29
Hemoglobin	5.33	1.56-18.23	0.008
Creatinine	3.34	1.12-9.99	0.031

*NLR:* neutrophil/lymphocyte ratio, *PLR:* platelet/ lymphocyte ratio.

## Discussion

The aim of this study was to determine the relationship between RCC subtypes and the associated mortality and biochemical parameters. An additional aim was to analyze the multiphasic multidetector CT findings. Previous studies have revealed that inflammatory markers are associated with RCC prognosis [18]. The NLR is a systemic inflammation marker with a wide range of use, low cost, and easy accessibility [19–21], and previous studies have concluded that the NLR is a predictor of mortality in RCC patients [22, 23]. As far as we know, inflammatory markers, such as the NLR and PLR, have been studied in RCC patients after they were classified generally as metastatic or not. However, these studies were focused on clear cell and metastatic RCCs. Additionally, in our study, the relationships between the RCC histological subtypes and mortality, systemic markers, and multiphasic multidetector CT findings were investigated. Our results suggest that in patients with RCC, there is a statistically significant difference in the NLR between survivors and non-survivors.

The logistic regression model indicated that the NLR was a significant predictor for mortality. Similar to our findings, Ohno et al. concluded that high preoperative NLR values are a predictor indicating mortality in RCC [13]. However, unlike us, Jagdev et al. found that a high NLR is not a predictor of prognosis in RCC [24]. Pichler et al. inferred (different from us) that the preoperative NLR is a predictor for mortality only in non-metastatic clear cell RCC [25]. According to the median PLR values, the Kaplan-Meier survival curves were significantly different between the surviving and non-surviving groups. The role of the histological subtype (papillary vs. chromophobe) as an independent prognostic factor in localized RCC, however, is still unclear.

In the literature, there were several studies including patients with clear cell RCC and the subtypes [13, 24–26]. However, there were no detected significant differences in mortality between the histological subtypes in our study. Dirican et al. [27] found no association between age, platelet count, neutrophil count, PLR, and mortality in metastatic RCC, except in lung metastasis. We found a significant difference between age, platelet count, neutrophil count, PLR, and mortality in metastatic and non-metastatic RCC.

In addition, we investigated the association between multiphasic multidetector CT findings and the other parameters in RCC. It is noted that mortality increased with an increased size of the lesion. Additionally, a significant difference with reference to CT density was determined in the evaluation of the lesion density between RCCs subtypes in corticomedullary and nephrographic phases. Similarly with Young et al. [28], significant difference between clear cell and papillary subtype in terms of lesion density on corticomedullary and nephrographic phase was detected. However, we didn’t identify difference on excretory phase. In our study, lesion density on corticomedullary phase was siginificantly higher in the dead patients compared with alive patients. Also, it is understood that there is significant difference between the RCC subtypes on corticomedullary phase and mortality effect this. Unlike Young et al. siginificant difference was detected between clear cell and sarcomatoid; papillary and mixed papillary; papillary and sarcomatoid subtypes. Besides, Young et al. calculated the average of two density values obtained from the two separate areas of the lesion. However we have the average density values of three different homogeneous enhanced areas. Unlike Jung et al. [29], attenuation values obtained on corticomedullary and nephrographic phases for clear cell were significantly higher than the non-clear cell ones. Again, different from Jung et al. our patient population was higher and additionally mixed and sarcomatoid types were included in our study. Also, differently, four phased CT examination was obtained. Jung et al. didn’t detect density differences in terms of gender. In our study, there was significant difference detected between male and female in terms of lesion density on the late phase. Unlike Kim et al. [30] there was statistically significant difference for lesion density on corticomedullary phase between clear cell and papillary; clear cell and sarcomatoid; papillary and mixed; papillary and sarcomatoid subtypes. On nephrographic phase in terms of lesion density, significant difference was detected between clear cell and papillary; papillary and mixed subtypes.

Our study is retrospective so it has some limiting factors. The pathological evaluation was performed by two different pathologists, and some prognostic factors, such as tumor necrosis, was not taken into consideration. Furthermore, the organs which the RCC metastasized to were not classified in and of themselves. In the evaluation of biochemical markers, only the preoperative period was taken into consideration, but the postoperative values were not included in the study. Clear cell RCC occurs more often than the other subtypes, and studies in which the clear cell and other subtypes are distributed homogeneously will provide more optimal results.

## Conclusions

As a result of this study, the NLR and PLR were determined to be easily accessible markers in the prognosis of RCC, and the usage of these markers may be helpful in the management of high-risk RCC patients. In addition, multidetector CT is useful to detect the differences in contrast enhancement patterns between the RCC subtypes.

## Abbreviations

CT: Computed tomography
EDTA: Ethylenediaminetetraacetic acid
IQR: Interquartile range
NLR: Neutrophil-to-lymphocyte ratio
PLR: Platelet-to-lymphocyte ratio
RCC: Renal cell carcinoma
ROI: Region of interest.
